# Could the Presence of Thrips AFFECT the Yield Potential of Genetically Modified and Conventional Maize?

**DOI:** 10.3390/toxins14070502

**Published:** 2022-07-19

**Authors:** Ľudovít Cagáň, Peter Bokor, Oxana Skoková Habuštová

**Affiliations:** 1 Faculty of Agrobiology and Food Resources, Slovak University of Agriculture, Trieda A. Hlinku 2, 949 76 Nitra, Slovakia; ludovit.cagan@gmail.com (Ľ.C.); peter.bokor88@gmail.com (P.B.); 2 Institute of Entomology, Biology Centre, Czech Academy of Sciences, 370 05 České Budějovice, Czech Republic

**Keywords:** Bt maize, non-target arthropods, abundance, Thripidae, pest control

## Abstract

Maize pests like *Ostrinia nubilalis* and *Diabrotica virgifera virgifera* are eradicated using genetically modified maize. This study’s goal was to see if the genetically modified maize MON810 is also toxic to thrips communities on maize. The impact of Bt maize on thrips diversity and abundance, as well as yield losses, was studied in the field in Borovce for three years (Slovakia). The study used 10 Bt and 10 non-Bt maize cultivars. Thrips were monitored every two weeks during the season using transparent sticky traps installed on the experimental plots (one per plot, 20 per year). In total, 3426 thrips were caught. Thrips populations usually peak around the end of July at BBCH55. Among the species identified were *Limothrips denticornis*, *Limothrips cerealium*, *Haplothrips aculeatus*, *Frankliniella schultzei*, *Frankliniella occidentalis*, *Thrips tabaci*, *Aeolothrips fasciatus*, *Frankliniella tenuicornis*, and *Chirothrips* spp. We found that MON810 maize had no effect on the occurrence or composition of thrips. Their presence was affected by the maize growth phase and growing seasons and partially by the weather. The direct effect on the grain yield was not confirmed. Our research contributed to scientific knowledge of thrips communities found on maize plants in Central Europe, including Bt maize.

## 1. Introduction

The transfer and expression of defense genes of the crop species are required for transgenic techniques in order to protect crops against pests. Those based on the application of *Bacillus thuringiensis* (Berliner, 1915) delta-endotoxin are among the most well-known and investigated examples of induced resistance, also known as Bt crops [1,2].

*Bacillus thuringiensis* Cry toxins are used in the control of insect pests [3]. The expression of the Cry1Ab gene allows the Bt maize to produce proteins that are highly toxic to some coleopteran and/or lepidopteran and to protect itself from significant insect pests common in European and North American agriculture (e.g., European corn borer), but considered harmless to vertebrates and another organism [3,4,5].

The commercialization of genetically modified crops has necessitated an evaluation of the technology’s potential environmental impacts, with non-target organisms (beneficial insects, natural pest controllers, rhizobacteria, growth-promoting microbes, and pollinators) among the most likely negative consequences [2,6]. When used as part of the overall integrated pest management (IPM) plan, Bt crops can aid in more effective biological control of both target and non-target pests [7]. Herbivores can be harmed by a toxin if they eat an insect-resistant transgenic plant directly [8,9], but comparative results of different experimental trials on Bt crop risk assessments in non-target insects show that Cry toxins used in transgenic crops are safe for the environment and have no lethal impact on biodiversity [6,10,11].

Despite the fact that thrips are common in maize fields [12], they are frequently overlooked because they do not generally cause serious damage to maize plants and thus are not considered major pests on this crop [13,14]. They are polyphagous by nature, colonizing maize for nearly the entire growing season [15]. Thrips are a relatively unstudied group of corn pests due to their small size and cryptic behavior. In Slovakia, the harmfulness of thrips on maize plants has received less attention than in Poland, where studies on their effect on corn plants were conducted in the 1950s and 1960s [16]. Thrips are important fodder for corn pests, according to Lisowicz [17], and they must be controlled. It was discovered also, that thrips actively take Cry toxins from genetically modified maize plants. This discovery placed them in an important group in the tritrophic structure in the maize ecosystem [18,19]. The toxin may be sucked by predators or parasitoid larvae feeding on thrips. Thrips damage can be caused directly by feeding on leaves, flowers, or fruits, resulting in white or silver stains on the leaves and ears caused by the cell contents being sucked out [20,21,22,23], or indirectly by viral transmission, pathogen infections, product contamination, and yield reduction [21,24,25,26]. Therefore, they are a serious pest of vegetable plants cultivated in the field and greenhouses where this piercing-sucking cosmopolitan insect serves as both pests and tospovirus vectors, spreading bacterial and fungal diseases and causing considerable economic damage in many regions of the world [15,20,21]. 

As Thripidae are weak fliers, they spread predominantly through the wind with the host plant seeds (anemochorically). Šefrová [27] corroborated in her study that the thrips were detected at 4500 m above the ground as a part of the aeroplankton. The temperature has a significant impact on the pace of thrips population growth. During the growing season, high temperatures promote population expansion, while low temperatures (cold) influence mortality and shorter generation times [28,29]. In a well-planned IPM strategy, pest population monitoring by employing sticky traps to collect insects has become an important decision-making tool [30,31]. 

Thrips on maize monitoring revealed that the abundant presence of thrips in south-eastern Poland occurred between the end of June and mid-August, depending on the research year, with a single population peak in the second half of July [32]. According to another author [16], the population peak of thrips on fodder corn occurs around the end of July or the beginning of August. The population peak of Thysanoptera on the plants was observed by Kucharczyk et al. [33] in the third week of July (2007) and early August (2006). The population dynamics of *F. occidentalis* thrips were determined by the growth phase of maize plants. Their abundance peaked in July 2003 and 2005, although due to later planting, the peak was shifted to the beginning of August 2004 [34]. Previous and current research has shown that thrips stop feeding in the second or third week of September, when the plants begin to dry down, regardless of corn type [32,33].

According to Cagáň et al. [35], the most common cereal thrips discovered in maize are *Limothrips denticornis*, *Haplothrips tritici*, and *Haplothrips aculeatus*. *Frankliniella tenuicornis*, *H. aculeatus* and *H. tritici* have all been identified as “cereal thrips” in Poland [36], however Zawirska [37] has identified two of these as maize pests. *Limothrips cerealium*, *L. denticornis*, and *Thrips angusticeps* are the most prevalent cereal thrips in Sweden, according to Larsson [28], whereas *L. cerealium*, *L. denticornis*, *H. aculeatus*, and *Anaphothrips obscurus* were also common in maize in Finland. In the Czech Republic, *Frankliniella occidentalis* has dominated on maize [38].

Over the duration of a year, researchers studied the spatiotemporal dynamics of thrips in cereals and discovered that numerous species of thrips interact with the area of various cultivated cereals [28]. This information is important, especially because thrips can be numerous in maize and, as a result of the global rise in the acreage of genetically modified maize [39], their harmfulness may increase.

Since Bourguet et al. [40] reported inconsistent results when comparing the abundance of thrips in Bt and non-Bt maize fields, the verification of the impact of Bt maize on thrips performance has become more important.

Obrist et al. [41] used life-table parameters to show that thrips grown on Bt maize were unaffected. They observed the effect of Bt on the thrips *F. tenuicornis* when it was fed on Bt and non-Bt maize. It was found that the risk to predators, when feeding on thrips in or near Bt maize fields, is negligible.

Furthermore, Malchau [42] stated that none of the life-table metrics tested for thrips reared on Bt and non-Bt maize was found to change. For example, some of the values (such as the number of eggs per female and lifespan) were lower than those reported in cereal-based experiments (wheat and oat).

In Slovakia, a three-year study was conducted using transparent sticky traps to monitor non-target insect species in Bt and non-Bt maize. Thrips were the most abundant species we discovered. We decided to try to collect data on the occurrence of thrips on maize plants in Slovakia because they are lacking. 

As a result, the goal of our study was to determine the difference in the abundance of thrips monitored by transparent sticky traps dependent on weather conditions and growth phases of maize (BBCH) installed in Bt and non-Bt maize plots, as well as crop losses, for three consecutive years, and to define their occurrence and species identification. The findings on thrips communities on maize plants, including Bt maize, in Slovakia, will add to scientific knowledge in Central Europe and could be used for future research.

## 2. Results

### 2.1. Occurrence of the Thrips in Maize Plots 

We monitored the presence of thrips on maize plants for three years via transparent sticky traps (2013–2015). We discovered nine different thrips species. From three years of monitoring, 3426 individuals were confirmed in the traps, with 1705 on non-Bt and 1721 on Bt maize. 

The abundance of thrips fluctuated annually over the three-year research. The year 2013 was the most numerous of all monitored experimental seasons (Table 1). The trapped thrips accounted for 1778 individuals, with 882 on non-Bt and 896 on the Bt maize plants. *Haplothrips aculeatus* was the most common species in the studied year, accounting for 22.44% of non-Bt plants and 24.80% of Bt plants among the total number of thrips detected. With a prevalence of 9% on both non-Bt and Bt maize plants, *Aeolothrips fasciatus* was the second most widespread species. Out of the total number of individuals captured in a given year, *F. tenuicornis* was the third most common species detected, accounting for 4.95% of the incidence on non-Bt maize plants and 4.72% on Bt maize plants (Table 1). 

The lowest number of thrips recorded during the monitored year was in 2014, with 430 individuals captured. Out of the total number of individuals captured in that year, *L. cerealium* was the most common species, accounting for 19.77% of non-Bt and 24.19% of Bt maize plants. *Haplothrips aculeatus* and *Frankliniella schultzei* were the second and third most abundant species, respectively, representing a similar incidence for non-Bt and Bt maize plants, although in reverse order (11.40% on non-Bt maize plants, 7.44% on Bt maize plants, 7.67% and 10.93% on Bt maize plants) (Table 1). 

The second, more frequent occurrence was confirmed in 2015, when 1 218 thrips were captured, 600 on non-Bt maize plants and 618 on Bt maize plants. *Limothrips cerealium* was the most common species, accounting for 20.85% and 22.5% of the species trapped in sticky traps in non-Bt and Bt maize plants, respectively. At the same time, after the *H. aculeatus* occurrence in 2013, this species represented the second most common group of thrips captured from the maize field over three years of monitoring. 

*Frankliniella schultzei* was the second most common species, accounting for 4.11% of non-Bt and 11.00% of Bt maize plants, respectively. *Frankliniella occidentalis* was found to be the third most abundant species, accounting for 9.28% of non-Bt plants and 4.52% of Bt plants among the total number of thrips captured that year. The remaining collected thrips (*L. denticornis*, *Thrips tabaci*, and *Chirothrips* spp.) also lived on non-Bt and Bt maize plants but were less common (see Table 1). The differences between the years 2013 and 2014 were not confirmed (*p* = 0.0533) (Table 2).

### 2.2. The Impact of Weather and Maize Physiological Phase on Thrips Occurrence

The prevalence of thrips varied from year to year. In the first year of monitoring, nine transparent sticky trap installations in maize plots were implemented. The dates for installing sticky traps were chosen to track thrips activity throughout the season in both non-Bt and Bt maize plots. The exhibit was designed around maize’s physiological phase (BBCH) and weather conditions. 

**Figure 1:**
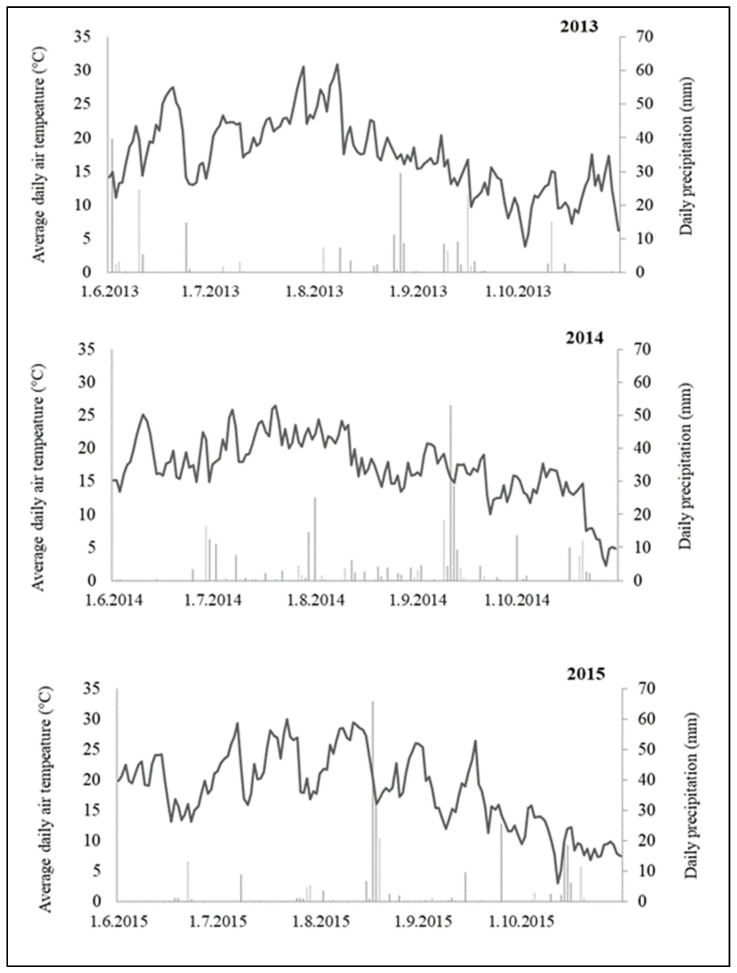
The curve represents the average daily air temperature (°C) and the columns daily precipitation (mm) during vegetation period 2013–2015. Locality: Borovce, Slovakia.

**Figure 2:**
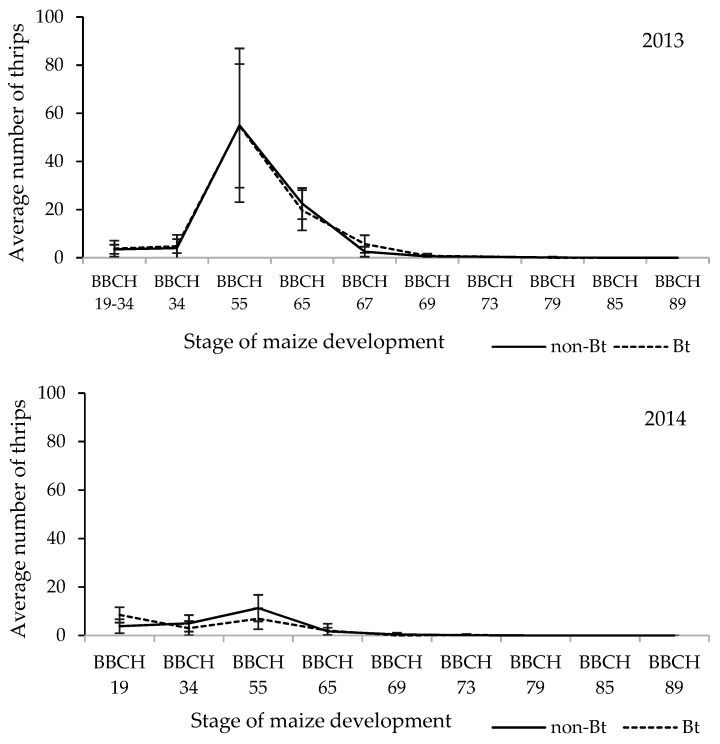

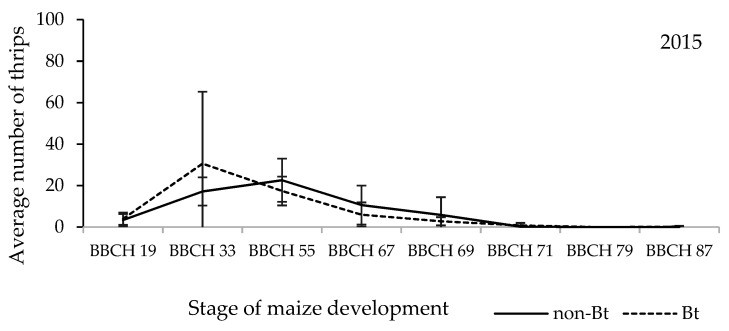
Number of thrips (seasonal distribution) monitored by sticky traps installed on non-Bt and Bt maize cultivar in field experiment during 2013–2015. Locality: Borovce, Slovakia. Averages from 10 repetitions. Each repetition is the sum of thrips from one transparent sticky trap. *X*-axis: BBCH-phenological growth phase of maize according to the identification keys [43]. BBCH 19–34 (9 or more leaves unfolded, 4 nodes are detectable). BBCH 55 (the center of the tassels begins to flower); BBCH 65 (Female: tips of stigmata visible, Male: upper and lower parts of tassel in flower); BBCH 67 (end of flowering); (BBCH 69–73 (end of flowering—milk stage of grain); BBCH 79 (Nearly all kernels have reached final size); BBCH 85 (Dough stage: kernels yellowish to yellow (variety dependent), about 55% dry matter); BBCH 89 (full-grain ripeness).

The initial distribution of sticky traps in a monitored year occurred at the beginning of the second half of June when the BBCH was between 19 and 34 (nine or more leaves unfolded, four nodes detectable). The thrips species *L. cerealium* and *F. tenuicornis* were found in roughly similar quantities at the start. Other species of thrips were only detected in small quantities (Table S1). At the time of trap placement, the weather was warm (Figure 1, Table S2). One peak of maximum thrips occurrence was documented in mid-July in the growth phase of maize BBCH 55 (the center of the tassels begins to flower) in the observed year (Figure 2). 

The last most abundant collection appears at the end of July (BBCH 65-female: stigmata tips visible, male: upper and lower parts of tassel in flower), with *H. aculeatus* taking first place, followed by *A. fasciatus*, *Chirothrips* spp., and *F. occidentalis*.

In August (BBCH 69–73, end of flowering—milk stage of grain), thrips numbers decreased with the exception of a few isolated pieces. Due to rising temperatures and precipitation, as well as frequent wind gusts, the thrips on maize plants decreased in the first and second part of August (BBCH 69–73, end of flowering—milk stage of grain) (Figure 1). By the end of the monitoring period (first week of October BBCH 89—full grain ripeness), no further gradation of thrips was observed (Figure 2, Table S1). 

Climate conditions factors observed before and after sample collection using the Pearson correlation coefficient were not confirmed significantly (Table 3), but their effect on thrips occurrence was obvious. Furthermore, statistical analyses revealed no statistically significant difference in thrips occurrence between Bt and non-Bt maize (*p* = 0.9861) (Table 2).

The year 2014 was the second year of monitoring with the same number of sticky traps installed as in the previous year. In comparison to the other two monitoring years, this year had the lowest number of thrips captured (Table 1). Statistical analyses found no significant differences between monitoring years (*p* = 0.053, Table 2). Thrips abundance appeared in two peaks of maximum occurrence. The first peak appears in the first week of June when the growth phase of maize was between BBCH 19 and 34 (nine or more leaves, four detectable nodes), and normal weather conditions prevailed (Figure 1 and Figure 2).

*Limnothrips cerealium*, *F. schultzei*, and *F. tenuicornis* were among the most numerous species captured by sticky traps on both (non-Bt and Bt) maize plants, but their occurrence differed. Thrips occurrence on Bt and non-Bt maize levelled out and were similar till the end of the monitoring period (BBCH 35 to BBCH 89-5 nodes detectable: full-grain ripeness) (Figure 2). The second peak of the most numerous species occurred during BBCH 55 (the tip of the tassel is visible) at the beginning of July, when nine thrips species were recorded. The second half of June had almost no precipitation, which contributed to a higher thrips abundance in the first week of July (Figure 2).

The occurrence of thrips was lowest in the second peak compared to both monitored years, and the most numerous species were: *L. cerealium*, *H. aculeatus*, *F. schultzei*, *F*. *tenuicornis*, and *A. fasciatus*. Less common species included *F. occidentalis*, *Chirothrips* spp., *T. tabaci*, and *L. denticornis* (Table 1).

Subsequent increases in temperature and precipitation led to a gradual decrease in the occurrence of thrips on maize plants during the growth phase of maize BBCH 65–67 (female stigmata fully emerged end male fully flowering—male flowering completed, female stigmata drying) in the second half of July and August. As in the previous year of monitoring, thrips did not occur at all at the end of the monitoring season (BBCH 89—full-grain ripeness (Figure 2).

The year 2015 was the final year of monitoring of the occurrence of thrips in field conditions using sticky traps distributed eight times on maize plants from BBCH stage 19 to BBCH 87 (nine or more leaves developed, physiological maturity). In this year, like in the previous years, two peaks of the most abundant species occurred (Figure 2). The first was recorded on Bt maize plants at BBCH 33 (three detectable nodes), and the second on non-Bt plants at BBCH 55 (the center of the tassels begins to flower).

The first thrips capture occurred in the second half of June (BBCH 19) when the presence of eight species of thrips was recorded. Limnothips cerealium was the most abundant species, occurring on both Bt and non-Bt maize, followed by *F. schultzei*, *F. tenuicornis*, *A. fasciatus*, and the remaining species represented only by individuals as *L. denticornis* and *T. tabaci*. The capture occurred during a period with relatively high average daily temperatures and a low precipitation profile (Figure 1, Tables S1 and S2). 

### 2.3. The Impact of the Occurrence of Thrips on the Yield of Maize 

In the first year of monitoring (2013), maize grain was harvested at the BBCH 90 growth stage of maize, when grain harvest moisture ranged from 25.9 to 29.7% for non-Bt maize and 27.1 to 29.4% for Bt maize. 

At a recalculated 14% humidity, the average maize grain yield on non-Bt and Bt plots was 6.44 and 6.08 t. ha^−1^. The harvest of maize grain was performed at BBCH 90 in the second year of monitoring (2014) at the same physiological stage of maize. In comparison to the first year, at a time when the harvest moisture of the grain was lower the second year it ranged from 20.3 to 21.5% for non-Bt maize and 16.5 to 21.1% for Bt maize. 

The average maize grain yield on non-Bt and Bt plots at 14% humidity was 1.66 and 1.82 times higher than in the first year of monitoring for the same plots, reaching 10.67 and 11.04 t. ha^−1^. In the third year of monitoring (2015), maize grain was harvested on experimental plots at BBCH 89, when the harvested moisture was the lowest compared to the previous two years, ranging between 16.6–18.2% for non-Bt maize and 16.6–17.7% for Bt maize. The obtained average maize grain yield at 14% humidity on non-Bt and Bt maize plots was the lowest in comparison to all monitored years, reaching 5.73 and 6.03 t. ha^−1^. 

We confirmed the significant influence of the experimental seasons between monitored years (*p* < 0.0001) using two-way ANOVA analyses. However, using Bonferroni’s multiple comparisons test, the significant differences in maize grain yield were obtained between 2013 and 2014 (*p* < 0.0001), 2013 and 2015 (*p* = 0.0222), and 2014 and 2015 (*p* < 0.0001) were confirmed (Table 2). The differences between maize grain yield obtained on Bt versus non-Bt plots were not confirmed (*p* = 0.3323).

## 3. Discussion 

The results demonstrate that thrips were not adversely affected by Bt maize when occurring on transgenic plants. This is consistent with another study in which thrips *F. tenuicornis* was unaffected when reared on Bt maize and the persistence of the Cry1Ab toxin in adult *F. tenuicornis* was short, resulting in a 97% decrease within the first 24 h [41]. Habuštová et al. [34] discovered no differences in *F. occidentalis* populations on Bt maize expressing Cry1Ab toxin and non-Bt maize. Bt maize expressing Cry3Bb1 toxin from *Bacillus thuringiensis* confers resistance to corn rootworms (*Diabrotica* spp.) and tolerance to the herbicide glyphosate-MON88017 demonstrates similar thrips insensitivity [44]. 

Bt crops have a narrow spectrum of activity, they have no negative effects on non-target organisms [45]. A novel *B. thuringiensis* (Bt)-transgenic toxin, Bt Cry51Aa2.834_16, on the other hand, is expected to reduce the need for insecticide applications targeting thrips in cotton [46]. So far, no studies on the population dynamics of thrips on maize have been conducted in Slovakia. Thrips were found in the maize field in the first half of June in our study, and the pest was abundant in south-western Slovakia between the end of June and the middle of July. Bereś et al. [16] discovered the greatest number of thrips between the first and third ten days of July, with population peaks on 12 July 2010; 20 July 2011; and 11 July 2012. Thrips were most abundant in sweet maize grown in south-eastern Poland between the end of June and the first ten days of August, with a population peak in the second ten days of July [47]. In previous studies conducted in Krzeczowice (south-eastern Poland) between 1982 and 1993, Lisowicz [32] reported that the population peak of thrips on maize was usually at the end of July or mid-August, and it occurred on 15 July 1993, which is similar to our study. Kucharczyk et al. [33] discovered that the population peak of thrips on maize usually occurs at the end of July or the first half of August 2006–2007, at the same location. Weather conditions in individual study years could have influenced thrips development dynamics (Figure 1). The temperature has the greatest influence on the life cycle of thrips. Higher temperatures shorten the life cycle, whereas temperature reductions can inhibit the development of pre-adult stages and thus lengthen the cycle [23,48]. Previous research has also shown that temperature has a positive effect on thrips populations on various crops, the population density rises with increasing temperature [48]. The developmental rate is also known to be a nonlinear function of temperature [49,50,51].

Linear approximation for this function may result in underdevelopment at low temperatures and overdevelopment at high temperatures. Additionally, changes in variability (e.g., diurnal range, frequency of extremes) can have a significant impact on insect populations [52,53]. Temperatures above 30 °C, on the other hand, can cause significant mortality in larvae and pupae [54]. Dintenfass et al. [55] discovered that in extreme high-temperature regions, the thrips population can be slowed by high temperatures and low relative humidity.

Similarly, some authors [56,57] reported that high temperatures and drought are detrimental to thrips survival, resulting in population decline. High daily temperatures, often exceeding 35 °C, most likely caused a significant decline in the thrips population and low occurrence during August and September in 2013–2015. (Figure 1). This was confirmed by the author [58], who reported that in Brazil, high temperatures and a lack of rainfall increased *T. tabaci* population density in garlic. When temperatures were between 18 °C and 21 °C and rainfall was low (114 to 144 mm per month), the most thrips were found. Another study [59] found that *T. tabaci* females laid the most eggs and lived the longest at temperatures ranging from 21.1 °C to 23.6 °C and relative humidity of 52%.

Bereś et al. [47] found a positive correlation between temperature and the number of adults *F. tenuicornis* and *H. aculeatus* individuals not only throughout the study period, but also from the time of plant infestation to the peak of pest population on sweet corn.

The thrips population on sweet corn peaked between 13–15 July 2008–2010, regardless of weather conditions, when plants were shed-ding pollen. In three years, we observed an increase in thrips population at the start of the vegetation period, and the peak of thrips population on maize occurred similarly on 15 July, 8 July and 16 July 2013–2015. The decrease in thrips populations in our study was most likely caused by pollen shedding and climatic conditions.

Contrary to expectations, changes in precipitation during the study periods had no significant effect on the number of thrips found on maize. The findings are consistent with earlier reports by Bereś et al. [47], who stated that analysis of individual cases revealed the precipitation may influence the number of thrips at the stage of plant infestation and population increase. According to Pierre and Dedryvér [60], rainfall and wind are frequently important factors in pest densities. Rainfall had an impact on thrips populations both ways positively and negatively. It has the potential to suppress thrips populations by killing larvae and adults. Rainfall also reduces thrips dispersal by inhibiting flight [61].

Other researchers discovered a significant negative correlation between thrips population and rainfall [62,63]. Rainfall, on the other hand, can have a positive impact on thrips populations by promoting plant growth and increasing pupal survival. Heavy rain has been reported to wash thrips off plants and down to the soil surface, resulting in significant population declines [64]. Although heavy rains are known to cause *T. tabaci* mortality [65] and thrips dispersal, light rain is unlikely to have contributed to the reduction in *T. tabaci* flight in the days afterward [64].

*Thrips tabaci* densities increased significantly as the temperature rose above 17 °C, with 90% of the thrips captured between 20.8 °C and 27.7 °C; no thrips were captured above 30.6 °C. *Thrips tabaci* densities on aerial traps decreased significantly as the wind speed increased, with no thrips captured at winds greater than 3.8 ms^−1^ [64]. We discovered no link between wind speed and the number of thrips caught in traps. Wind speed was usually greater than 5 ms^−1^ during the weeks when traps were installed in maize fields (Table 3).

The presence of thrips, monitored by sticky traps placed on maize plants at various stages of growth, may have an impact on the maize grain crop. Our findings are consistent with the findings of a study [66], which concluded that the impact of thrips on maize plants observed in the south-eastern part of Poland had an indirect effect on plant damage and crop reduction, and their effect was minimal. 

In our case, the highest maize grain crop was obtained in 2014, which also had the lowest occurrence of thrips. The Bonferroni’s multiple comparison test, however, revealed highly significant differences in maize grain yield between 2013 and 2014, and 2014 and 2015. Significant differences in maize grain crops were also found between 2013 and 2015, when there was a high abundance of thrips on the plants. The negative effect of thrips on maize grain yield obtained on Bt versus non-Bt plots was not confirmed. As a result, we must assume that the effect of thrips monitored by sticky traps in our case was only marginal and had no significant impact on maize yield reduction. 

## 4. Conclusions

During the study years, nine species of thrips were found on the maize plants, which represented 3 426 individual thrips. Thrips began infesting the maize plants at the stage BBCH 19–34 and ceased infesting plants in BBCH 69. The population peak usually occurred at the end of July (BBCH 55). The species included *Limothrips denticornis*, *Limothrips cerealium*, *Haplothrips aculeatus*, *Frankliniella schultzei*, *Frankliniella occidentalis*, *Thrips tabaci*, *Aeolothrips fasciatus*, *Frankliniella tenuicornis* and *Chirothrips* spp. MON810 maize did not adversely affect the number or composition of thrips. Climate conditions factors observed before and after sample collection using the Pearson correlation coefficient were not confirmed significantly, but their effect on thrips occurrence was obvious. 

Furthermore, maize grain yield, abundance, and seasonal dynamics of the followed thrips on plants were not affected by the type of maize grown (Bt versus non-Bt) but were affected by the growing season. The differences in the monitored years were not confirmed, but the occurrence of the thrips varied depending on the time of sampling collection. We confirmed the significant influence of the experimental seasons on maize grain yield between the monitored years 2013 and 2014, 2013 and 2015, and 2014 and 2015. Our obtained data also provide information about the thrips species representations typical for the cereals, represented on Bt and non-Bt maize plants and their seasonal dynamics which can be useful for future planning of maize integrated plant protection in Central Europe if thrips started to be a serious pest of maize. 

Climatic conditions changed during the study; we do not confirm their significant differences but their effect on thrips occurrence was obvious; more research is needed to confirm or refute their effect on the presence of thrips on maize plants.

## 5. Materials and Methods

### 5.1. Experimental Site and TRIAL Design

During the years 2013–2015, a field trial was conducted in the village of Borovce in western Slovakia. The coordinates of the site were 48°34′ N, 17°43′ E, with altitude of 181 m a.s.l. and an Udoll soil. Winter wheat was the preceding crop in all three years of the study. Sowing dates varied according to weather conditions, with 9 May 2013; 28 April 2014; and 5 May 2015.

In 2013, the field was fertilized with urea (130 kg/ha: N—46%) and Polidap (200 kg/ha: P—46%; N—18%) before sowing and treated by herbicides Maister (0.15 kg/ha; active ingredient: iodosulfuron-methyl Na-1-gthiencarbazone-methyl-10-gforamsulfuron-30 g) and Istroekol (adjunctive substance 2.0 l/ha) on 26 May. The experimental area was fertilized in 2014 with the same preparations as the previous year, but with a different N dose (100 kg/ha: N—46%). Herbicides Dual Gold (active ingredient: S-metolachlor 960 g/L; 1.25 kg/ha) on May 7, and Mustang (active ingredient: aminopyralid 10 g/L + florasulam 5 g/L + 2.4 D 180 g/L; 0.8L/ha) on 26 May were applied after sowing. In 2015, the experimental area was fertilized before sowing with urea in the same dose as in 2014, but NPK 15-15-15 in dose (150 kg/ha: P—22.5%; N—22.5%; K—22.5%) was used. Herbicide Wing P (4.0 L/ha) was applied after sowing on 7 May.

Maize hybrids were sown in 10 replicated plots of 10 m × 10 m each. A 5 m wide strip of barley separated each plot from the others. The trials for the Bt and isogenic plots were completely random. The experiments were carried out with identical plot arrangements during all three vegetation seasons. DKC3872YG (Bt maize line MON810) and its near-isogenic non-Bt maize line DKC3871 were used in the experiment. Monsanto Company supplied the seeds (MONSANTO Technology LLC, St. Louis, MO, USA). The maize grain was harvested based on the physiological growth phase of maize (BBCH) and the weather.

Maize was harvested at BBCH 90 (fully ripe: kernels were hard and shiny, and leaves began to turn a yellow color) in 2013 and 2014, but in 2015 at BBCH 89 (fully ripe: kernels hard and shiny, leaves still green). In both cases, the grain was harvested at the peak of its ripeness. The maize grain was collected using a CLASS LEXION 600 harvester with an adjustable row adapter. The harvested maize grain from each experimental plot was weighed, and the harvested moisture was measured. The maize grain moisture was determined using the HE 50 BT operating grain hygrometer. A maize husk was taken from one plant, and the grain was crushed and poured into a moisture meter container for grinding. The container enclosing the grain powder was then placed in the hygrometer, with the measuring parameters for the maize grain and the current pre-harvest moisture determined. During the experimental years 2013–2015, weather conditions such as precipitation profiles, average temperatures, wind speed, and wind gusts were recorded. Changes in weather conditions are presented from June to October to cover the entire monitoring period (Figure 1, Table S2).

### 5.2. Data Analysis and Thrips Sampling 

Transparent sticky traps were used to monitor the abundance of thrips in the experimental area. The traps were placed every two weeks in the middle of the experimental plots on the upper part of the maize plant on Bt and non-Bt maize plots (1 trap per plot).

Traps were installed 8 or 9 times per season to capture all important growth stages of maize, and the period with the highest occurrence of thrips and collected for determination after a seven-day interval. Annually, 180 (2013, 2014) and 160 (2015) traps were used. In total 520 transparent sticky traps were analyzed for the presence of thrips depending on the physiological growth stage of maize. The presence of thrips over the three years of monitoring was related to maize yields and the effect on the maze yield was evaluated. The number of thrips in each trap was counted and identified to the species level using the keys of Cluever and Smith [67], Mound et al. [68,69], and Reed et al. [70]. 

Statistical analysis included calculating differences in trip occurrence (all years combined and each year individually) and differences in maize grain yield in each study year and between years (Bt vs. non-Bt plots) using two-way ANOVA and Bonferroni’s multiple comparisons test. The Pearson correlation coefficient between climatic conditions before and during thrips collection was also calculated for the entire monitoring period. 

## Figures and Tables

**Table 1:** The species number of thrips found on the maize plants during the growing period in three consecutive years. Numbers are the sum of 10 transparent traps collected after a seven-day interval.

Species/Genus	2013 (N)		2014 (N)		2015 (N)		2013 (%)		2014 (%)		2015 (%)	
	Non-Bt	Bt	Non-Bt	Bt	Non-Bt	Bt	Non-Bt	Bt	Non-Bt	Bt	Non-Bt	Bt
*Limothrips denticornis*	32	26	4	0	6	1	1.80	1.46	0.93	0.00	0.49	0.08
*Limothrips cerealium*	70	80	85	104	254	271	3.94	4.50	19.77	24.19	20.85	22.25
*Haplothrips aculeatus*	399	441	49	33	50	25	22.44	24.80	11.40	7.67	4.11	2.05
*Frankliniella schultzei*	63	38	32	47	50	134	3.54	2.14	7.44	10.93	4.11	11.00
*Frankliniella occidentalis*	32	34	9	1	113	55	1.80	1.91	2.09	0.23	9.28	4.52
*Thrips tabaci*	1	2	3	3	10	11	0.06	0.11	0.70	0.70	0.82	0.90
*Aeolothrips fasciatus*	161	160	13	5	49	52	9.06	9.00	3.02	1.16	4.02	4.27
*Frankliniella tenuicornis*	88	84	23	13	68	67	4.95	4.72	5.35	3.02	5.58	5.50
*Chirothrips* spp.	36	31	5	1	0	2	2.02	1.74	1.16	0.23	0.00	0.16
**The total amount of thrips**	**882**	**896**	**223**	**207**	**600**	**618**	**49.61**	**50.39**	**51.86**	**48.14**	**49.26**	**50.74**
	**1778**		**430**		**1218**		**100**		**100**		**100**	

(N)-number of specimens; % percentage share.

**Table 2:** The findings of a two-way Anova statistical analysis of thrips occurrence on Bt and non-Bt maize plants were monitored using transparent sticky traps and maize grain yield. The *p*-values that are statistically significant are highlighted. Bonferroni’s multiple comparisons test (*p*) ^1^ results are only provided for significantly different parameters.

Examined Parameter	Test Statistics F (DFn, DFd)	Bonferroni’s Multiple Comparisons Test (*p*) ^1^
**Occurrence of thrips** Bt vs. Non-Bt	F (1, 46) = 0.0003072; *p* = 0.9861	
Year of monitoring	F (2, 48) = 3.119; *p* = 0.053	
Date of collection	F (25, 468) = 41.69; ***p* < 0.0001**	Bt vs. Non-Bt: 1. 7. 2015 (BBCH 33); ***p* = 0.0089**
Months of collections	F (4, 90) = 197.9; ***p* < 0.0001**	Bt vs. Non-Bt: *p* > 0.9999 *
**Yield of maize grain** Bt vs. Non-Bt	F (1, 27) = 0.9746; *p* = 0.3323	
Year of harvesting	F (2, 27) = 864.7; ***p* < 0.0001**	2013 vs. 2014: ***p* < 0.0001** 2013 vs. 2015: ***p* = 0.0222** 2014 vs. 2015: ***p* < 0.0001**

* In total, there were no differences in thrips occurrences between Bt and non-Bt maize plots. The difference was proven only in the month of sample collection when the highest occurrence of thrips was confirmed in all years.

**Table 3:** Pearson correlation coefficient among climatic conditions during the collection of thrips and thrips numbers during monitoring seasons 2013–2015. T (+7) = average daily temperature during seven days of trap installation. T (−7 + 7) = average daily temperature during seven days before trap installation and during the time of trap installation. R (+7) = sum of precipitation during seven days of trap installation, R (−7 + 7)—sum of precipitation during seven days before trap installation and during the time of trap installation. R (−30 + 7) sum of precipitation during 28 days before trap installation and during the time of trap installation.

Followed Parameters	T (+7)	*p*-Value	T (−7 + 7)	*p*-Value	R (+7)	*p*-Value	R (−7 + 7)	*p*-Value	R (−30 + 7)	*p*-Value
Average No. of thrips on: Non-Bt 2013	0.355818	0.3473	0.35197	0.3529	−0.42231	0.2575	−0.46952	0.2022	−0.28568	0.4562
Bt 2013	0.364725	0.3345	0.37427	0.3210	−0.40955	0.2737	−0.46523	0.2070	−0.28983	0.4493
Non-Bt 2014	0.355418	0.3479	−0.07175	0.8545	−0.27904	0.4671	−0.08482	0.8282	−0.53828	0.1349
Bt 2014	0.351807	0.3532	−0.03245	0.9339	−0.37886	0.3147	−0.21572	0.5772	−0.47690	0.1943
Non-Bt 2015	0.172575	0.6828	0.11284	0.7902	−0.03377	0.9367	−0.27957	0.5025	−0.67649	0.0952
Bt 2015	−0.054100	0.8988	−0.33063	0.4238	−0.21553	0.6082	−0.18084	0.6682	−0.46047	0.2984
Non-Bt 2013–15	0.274293	0.1751	−0.06663	0.7464	−0.21733	0.2862	−0.27033	0.1817	−0.35888	0.0718
Bt 2013–15	0.222794	0.2740	−0.06597	0.7488	−0.22819	0.2622	−0.25954	0.2004	−0.32175	0.1090

## Data Availability

All published data are available within the article and Supplementary Materials online.

## Supplementary Materials

The following supporting information can be downloaded at: https://www.mdpi.com/article/10.3390/toxins14070502/s1; Table S1. Number of thrips in transparent sticky traps installed in maize field experiment during experimental seasons 2013–2015. Locality: Borovce, Slovakia. Sum from 10 repetitions. Each repetition is the sum of thrips from one transparent sticky trap that were collected after seven days of their installation. Non-Bt-(DKC 3871), Bt-maize (DKC 3872 YG). Table S2. Climatic data among climatic conditions during the collection of thrips in the experimental seasons 2013–2015. T (+7) = average daily temperature during seven days of trap installation. T (−7 + 7) = average daily temperature during seven days before trap installation and during the time of trap installation. R (+7) = sum of precipitation during seven days of trap installation, R (−7 + 7)—the sum of precipitation during seven days before trap installation and during the time of trap installation. R (−30 + 7) sum of precipitation during 28 days before trap installation and during the time of trap installation. WS (+7) hourly means of wind speed (ms^−1^) and WG (+7) wind gusts (ms^−1^) during seven days of trap installation.
