# Supplementary material for: Could the Presence of Thrips AFFECT the Yield Potential of Genetically Modified and Conventional Maize?
Source: Toxins (Basel). 2022 Jul 19;14(7):502. doi: 10.3390/toxins14070502 (PMC9320106; doi:10.3390/toxins14070502)
Supplement: Supplementary file 1 [file toxins-14-00502-s001.zip › toxins-1772282-supplementary.pdf]

toxins

MDPI

Article

Could the Presence of Thrips AFFECT the Yield Potential of Genetically Modified and Conventional Maize?

Ľudovít Cagáň, Peter Bokor and Oxana Skoková Habuštová

toxins

MDPI

Table S1. Number of thrips in transparent sticky traps installed in maize field experiment during experimental seasons **2013-2015**. Locality: Borovce, Slovakia. Sum from 10 repetitions. Each repetition is the sum of thrips from one transparent sticky trap that were collected after seven days of their installation. Non-Bt- isoline (DKC 3871), Bt - maize (DKC 3872 YG).

**Date of collection**	*Limothrips denticornis*		*Limothrips cerealium*		*Haplothrips aculeatus*		*Frankliniella schultzei*		*Frankliniella occidentalis*		*Thrips tabaci*		*Aeolothrips fasciatus*		*Frankliniella tenuicornis*		*Chirothrips spp.*	
	**Non-Bt**	**Bt**	**Non-Bt**	**Bt**	**Non-Bt**	**Bt**	**Non-Bt**	**Bt**	**Non-Bt**	**Bt**	**Non-Bt**	**Bt**	**Non-Bt**	**Bt**	**Non-Bt**	**Bt**	**Non-Bt**	**Bt**
**2013**																		
June 17	1	0	10	14	1	1	7	2	1	1	0	1	0	0	14	19	1	0
July 1	0	2	4	3	0	0	6	1	1	2	0	0	21	34	8	6	0	0
July 15	29	21	50	56	293	324	41	33	4	11	1	1	83	55	47	44	2	3
July 29	2	3	6	6	102	99	5	2	23	18	0	0	48	42	13	6	26	21
August 12	0	0	0	1	0	13	4	0	3	2	0	0	9	29	6	9	3	3
August 26	0	0	0	0	2	4	0	0	0	0	0	0	0	0	0	0	4	4
September 09	0	0	0	0	0	0	0	0	0	0	0	0	0	0	0	0	0	0
September 23	0	0	0	0	1	0	0	0	0	0	0	0	0	0	0	0	0	0
October 07	0	0	0	0	0	0	0	0	0	0	0	0	0	0	0	0	0	0
**2014**																		
June 10	0	0	21	47	0	0	11	31	0	0	1	1	0	0	5	6	0	0
June 24	1	0	20	17	2	1	8	4	3	0	1	1	6	2	9	4	0	1

July 08	1	0	40	37	38	20	10	6	6	0	1	1	7	2	9	2	1	0
July 22	2	0	4	3	9	10	0	4	0	1	0	0	0	1	0	1	2	0
August 05	0	0	0	0	0	2	2	0	0	0	0	0	0	0	0	0	2	0
August 19	0	0	0	0	0	0	1	2	0	0	0	0	0	0	0	0	0	0
September 03	0	0	0	0	0	0	0	0	0	0	0	0	0	0	0	0	0	0
September 16	0	0	0	0	0	0	0	0	0	0	0	0	0	0	0	0	0	0
October 01	0	0	0	0	0	0	0	0	0	0	0	0	0	0	0	0	0	0
**2015**																		
June 18	1	1	16	16	0	1	6	8	2	2	1	1	2	3	6	8	0	0
July 01	2	0	89	113	4	5	40	126	6	3	7	10	10	24	14	25	0	0
July 16	3	0	123	75	11	3	3	0	25	10	2	0	0	0	33	20	0	0
July 30	0	0	0	1	33	8	1	0	30	19	0	0	36	24	6	8	0	0
August 12	0	0	0	0	1	2	0	0	50	21	0	0	1	1	7	4	0	0
August 27	0	0	0	0	1	6	0	0	0	0	0	0	0	0	1	2	0	0
September 01	0	0	0	0	0	0	0	0	0	0	0	0	0	0	0	0	0	0
September 24	0	0	0	0	0	0	0	0	0	0	0	0	0	0	1	0	0	2

toxins

MDPI

Table S2. Climatic data among climatic conditions during the collection of thrips in the experimental seasons **2013-2015**. T (+7) = average daily temperature during seven days of trap installation. T (-7+7) = average daily temperature during seven days before trap installation and during the time of trap installation. R (+7) = sum of precipitation during seven days of trap installation, R (-7+7) – the sum of precipitation during seven days before trap installation and during the time of trap installation. R (-30+7) sum of precipitation during 28 days before trap installation and during the time of trap installation. WS (+7) hourly means of wind speed (ms^{-1}) and WG (+7) wind gusts (ms^{-1}) during seven days of trap installation.

Year	Date of trap collection	T (+7)	T (-7+7)	R (+7)	R (-7+7)	R (-30+ 7)	WS (+7)	WG (+7)
2013	June 17	19.725	18.09333	30.3	36.1	94.7	5.5714	9.5714
2013	July 01	14.5125	19.49333	15.8	15.8	103.0	6.5714	11.7143
2013	July 15	19.75	20.35333	3.6	5.4	51.5	5.7143	9.4286
2013	July 29	25.0875	23.21333	0.1	0.1	21.3	3.4286	6.5714
2013	August 12	24.6625	24.96667	10.9	18.4	22.1	5.0000	9.2857
2013	August 26	18.55	18.96667	14.5	20.1	35.0	5.7143	9.8571
2013	September 09	16.7125	16.80667	8.7	48.5	75.1	5.1429	8.8571
2013	September 23	12.675	13.58667	29.2	56.0	111.7	6.5714	10.2857
2013	October 07	8.75	10.4	0.0	0.1	56.3	5.8571	9.1429
2014	June 10	19.4125	16.96	0.3	15.5	66.5	3.1429	6.2857
2014	June 24	17.5	18.58667	0.0	0.7	21.5	4.8571	9.8571
2014	July 08	21.125	19.72	19.2	51.3	52.3	4.4286	8.7143
2014	July 22	23.6375	329.3	5.6	14.6	58.3	5.1429	11.2857
2014	August 05	22.2	21.76	41.6	51.5	74.7	5.2857	9.7143
2014	August 19	17.5625	19.8	11.3	15.0	69.1	6.1429	10.0000
2014	September 03	16.0375	15.96875	14.6	26.7	83.2	4.8571	7.5714
2014	September 16	16.95	18.02857	118.3	118.4	156.3	4.7143	8.4286
2014	October 01	13.5625	14.65625	14.9	21.3	154.3	4.2857	8.0000
2015	June 18	20.1625	20.36667	1.1	1.1	47.3	5.4286	10.4286
2015	July 01	18.3625	16.62	0	15.7	37.5	5.2857	9.8571
2015	July 16	19.9875	2.3	0.4	9.2	24.9	5.1429	11.2857
2015	July 30	21.475	24.03333	12.7	12.7	21.9	5.8571	10.4286
		27.5125	24.74286	0.2	3.7	16.8	3.5714	9.5714

toxins

MDPI

2015	August 12							
2015	August 27	19.0625	20.89333	4.4	131.0	147.4	5.0000	9.8571
2015	September 10	15.5125	19.38667	2.1	2.5	133.7	6.1429	10.1429
2015	September 24	17.175	17.84667	0.2	10.9	37.8	6.8571	11.5714
